# Feline and Canine Cutaneous Lymphocytosis: Reactive Process or Indolent Neoplastic Disease?

**DOI:** 10.3390/vetsci9010026

**Published:** 2022-01-11

**Authors:** Francesco Albanese, Francesca Abramo, Michele Marino, Maria Massaro, Laura Marconato, Lucia Minoli, Valeria Martini, Luca Aresu

**Affiliations:** 1 Laboratorio MYLAV, 20017 Milan, Italy; francescoalbanese@laboratoriolavallonea.it (F.A.); michelemarino@laboratoriolavallonea.net (M.M.); mariamassaro@laboratoriolavallonea.net (M.M.); 2 Department of Veterinary Sciences, University of Pisa, 56124 Pisa, Italy; 3 Department of Veterinary Medical Sciences, University of Bologna, Ozzano dell’Emilia, 40064 Bologna, Italy; laura.marconato@unibo.it; 4 Department of Veterinary Sciences, University of Turin, 10095 Turin, Italy; lucia.minoli@unito.it (L.M.); luca.aresu@unito.it (L.A.); 5 Department of Veterinary Medicine, University of Milan, 26900 Lodi, Italy; valeria.martini@unimi.it

**Keywords:** cat, cutaneous lymphocytosis, dog, immunohistochemistry, lymphoma, PARR, skin

## Abstract

Cutaneous lymphocytosis (CL) is an uncommon and controversial lymphoproliferative disorder described in dogs and cats. CL is generally characterized by a heterogeneous clinical presentation and histological features that may overlap with epitheliotropic lymphoma. Therefore, its neoplastic or reactive nature is still debated. Here, we describe clinicopathological, immunohistochemical, and clonality features of a retrospective case series of 19 cats and 10 dogs with lesions histologically compatible with CL. In both species, alopecia, erythema, and scales were the most frequent clinical signs. Histologically, a dermal infiltrate of small to medium-sized lymphocytes, occasionally extending to the subcutis, was always identified. Conversely, when present, epitheliotropism was generally mild. In cats, the infiltrate was consistently CD3+; in dogs, a mixture of CD3+ and CD20+ lymphocytes was observed only in 4 cases. The infiltrate was polyclonal in all cats, while BCR and TCR clonal rearrangements were identified in dogs. Overall, cats had a long-term survival (median overall survival = 1080 days) regardless of the treatment received, while dogs showed a shorter and variable clinical course, with no evident associations with clinicopathological features. In conclusion, our results support a reactive nature of the disease in cats, associated with prolonged survival; despite a similar histological picture, canine CL is associated with a more heterogeneous lymphocytic infiltrate, clonality results, and response to treatment, implying a more challenging discrimination between CL and CEL in this species. A complete diagnostic workup and detailed follow-up information on a higher number of cases is warrant for dogs.

## 1. Introduction

Canine and feline cutaneous lymphocytosis (CL) is an uncommon lymphoproliferative disorder, sharing clinical and histological similarities with the human counterpart, for which etiology and pathogenesis are not completely understood [1,2]. So far, different terms have been used to describe this entity in veterinary medicine, including cutaneous pseudolymphoma, lymphocytoma cutis, cutaneous lymphoid hyperplasia, and indolent lymphoma, thereby limiting prediction of clinical behavior [3]. Indeed, CL represents a diagnostic dilemma mainly due to the difficulties in distinguishing it from cutaneous lymphoma, thereby preventing the ability to anticipate prognosis.

In cats with CL, skin lesions progress over time, eventually spreading to the viscera, thus indicating a clinical behavior more similar to lymphoma rather than an inflammatory disorder [4,5]. The condition is predominately reported in old cats, without any breed predisposition and with a slight predominance in females [4]. Usually, the disease has an acute onset and progresses slowly [6]. The lateral thorax is more often affected, but any other anatomical site can be involved; systemic signs are rare. Cutaneous lesions are represented by alopecia, erythema, and scales with or without ulcers and crusts [4,6].

In dogs, middle aged to old patients are predominately affected without any sex or breed predisposition. Axillae and groin are more commonly involved either with unilateral or symmetric distribution. Alopecia, erythema, macules, scales, and slightly raised plaques are the prevalent cutaneous lesions [5].

Recently, CL has been reclassified as an indolent lymphoma; however, progression to a high-grade lymphoma has been described [5]. Histologically, CL is characterized by a diffuse infiltration of small lymphocytes in the dermis with or without epitheliotropism. Nevertheless, in many cases it is challenging to differentiate between an inflammatory disorder and cutaneous lymphoma [4,5,7].

To date, chronic inflammatory disorders are mainly characterized by a diffuse homogenous lymphocytic infiltrate, while cutaneous epitheliotropic lymphoma (CEL), although varying among different forms, shows a peculiar tropism for epithelial structures of the neoplastic cells as a key diagnostic feature, and a varying degree of lymphocytic dermal infiltration, going from minimal and superficial to diffuse and nodular [7].

Unfortunately, PCR for antigen receptor gene rearrangements (PARR) does not always help to discriminate the origin of this disease [8]. While initially considered the gold standard to differentiate cutaneous reactive disorders (polyclonal) from CEL (clonal) [9,10,11], recent studies have revealed contradictory data including clonal populations showing unexpected spontaneous regression and polyclonal populations progressing to overt lymphoma, thereby questioning its clinical utility [7,12].

Due to the rarity of CL and the difficulty in predicting its clinical behavior, the aims of this retrospective study were (i) to describe clinical, histological, and immunohistochemical features of 19 feline and 10 canine cases histologically compatible with CL and (ii) to correlate PARR results with clinical data.

## 2. Materials and Methods

### 2.1. Animals

Formalin-fixed and paraffin-embedded skin biopsy samples from cats and dogs with a histopathological diagnosis of CL were retrieved from the pathology archives of MYLAV laboratory (Passirana di Rho, Milan, Italy) and the Department of Veterinary Sciences, University of Pisa (Italy). All specimens were obtained for diagnostic purposes with the informed consent of the pet owners. Minimal clinical inclusion criteria were as follows: species, breed, age, sex, number of lesions (single or multiple), anatomic site, time interval between lesion appearance and biopsy, and time to progression.

Additional data were collected by the referring veterinarians using a questionnaire, including clinical signs at diagnosis, staging, treatment, response, date, and cause of death. When available, staging included hemato-biochemistry, urinary assay, imaging (abdominal ultrasound, radiography, or computed tomography), lymph node, spleen, and liver cytology. Response was defined as “complete” when full regression of the lesions was achieved, “partial” when clinical signs were less severe but still present, “stable” when no progression neither amelioration of clinical signs was noticed despite treatment, and “progressive disease” when clinical signs were worsening despite treatment. Twenty-eight days were considered to define any of the aforementioned responses. Overall survival (OS) was defined as the interval between diagnosis and death from any cause, and calculated with the Kaplan–Meier method. Animals lost to follow-up were censored at the date of last contact.

### 2.2. Histopathology

For all cases, two independent operators, a dermatopathologist (F.Ab.) and a dermatologist with extensive experience in dermatopathology (F.Al.), reviewed 4–5 µm hematoxylin–eosin (H&E) cutaneous tissue sections. Inclusion criteria were the presence of a diffuse dermal infiltration of small to medium lymphocytes with or without epitheliotropism [4,5]. Discrepancies were solved by consensus and cases with doubtful interpretation by both examiners were excluded. All samples were investigated by scoring epidermal, dermal, adnexal structures histopathological alterations, and lymphocytic phenotype. In detail, the presence of crusts and ulcers in the epidermis was scored (0 = absence, 1 = presence). A semiquantitative score from 0 to 3 (0 = absence, 1 = mild, 2 = moderate, 3 = severe) was used to evaluate hyperkeratosis (either basket weave and compact type), parakeratosis, hyperplasia, spongiosis, inflammation, and epidermotropism. For dermis, a score from 0 to 3 was assigned for the distribution pattern (0 = superficial, 1 = mid dermis, 2 = deep dermis, 3 = perivascular and diffuse). Moreover, for the Grenz zone a score 1 was assigned when present and 0 when absent. In addition, the size of lymphocytes (small, medium, and large), the presence of both nuclear indentations and nodular lymphoid aggregates were recorded. For adnexal structures, the severity of intraepithelial lymphocytic infiltration (folliculotropism, syringotropism, and tropism for sebaceous glands) was scored from 0 to 3 (0 = absence, 1 = mild, 2 = moderate, 3 = severe). Mitotic count was assessed in 10 consecutive high-power fields (HPF) (400×).

### 2.3. Immunohistochemistry

Immunohistochemistry (IHC) was performed for all cases. Serial paraffin sections were processed using an automatic immunostainer (Lab Vision Autostainer 480S, Thermo Scientific, Waltham, MA, USA). Immunohistochemical analysis was carried out in accordance with the guidelines of the American Association of Veterinary Diagnosticians (AAVLD) Subcommittee on Standardization of Immunohistochemistry [13]. For both species, a monoclonal mouse anti-human CD3 antibody (dilution 1:200, Dako F7.2.38, Glostrup, Denmark) and a monoclonal rabbit anti-CD20 antibody (dilution 1:200, Abcam SP32, Cambridge, UK) were used for the identification of T and B lymphocytes, respectively. Antibody cross-reactivity with feline and canine antigens was confirmed by internal laboratory testing on lymphoid tissues and tumors from both species. Cytoplasmic CD3 and membranous CD20 positivity were considered specific immunoreactivity patterns. Sections of feline and canine hyperplastic lymph nodes were added to each immunohistochemistry run as positive controls. Negative controls were prepared by replacing the primary antibody with an irrelevant one. 

All samples were investigated for the presence of CD3+ and CD20+ lymphocytes infiltrating the dermis and scored as follows: 0 = absence or <10%, 1 = 10–49%, 2 = 50–89%, 3 ≥90%. The presence of CD3+ and CD20+ lymphocytes within nodular aggregates was also evaluated (0 = absence and 1 = presence).

### 2.4. PARR

PARR was used to detect clonal rearrangements of T-cell receptor gamma locus (TRG) and B-cell receptor (BCR). Briefly, total genomic DNA was isolated from previously deparaffinized and rehydrated three-micrometer-thick sections using the QIAsymphony DSP DNA Mini kit (Qiagen, Milan, Italy) following the manufacturer’s instructions. PCR was performed in a final reaction volume of 50 µL, in duplicate, containing 25 µL of 2× HotStarTaq Master Mix (Qiagen, Milan, Italy), 0.3 µM of each primer, and 5 µL of DNA template. The reaction was brought to the final volume of 50 µL with PCR water. The primer sequences for the canine clonality test were obtained by Burnett et al. 2003 [14]. For the feline clonal BCR and TCR rearrangements clonality test primer sequences were obtained by Mochizuki et al. 2011 [15] and Moore et al. 2005 [16], respectively. All primer sequences of both assays are reported in the Supplementary Materials Section (Supplementary Table S1). The PCR products were separated by capillary gel electrophoresis using the QIAxcel Advanced (Qiagen, Milan, Italy) and represented as electropherograms by the QIAxcel ScreenGel Software 1.5 (Qiagen). Results were deemed clonal when the electrophoresis profile showed one or more reproducible peaks, with the same height present in both reaction repeats [17].

## 3. Results

### 3.1. Clinical Presentation

From 2009 to 2020, 19 cats and 10 dogs with a histopathological diagnosis of CL have been included in the study. Patients’ data and clinical presentation are reported in Table 1 and Table 2.

**Figure 1:**
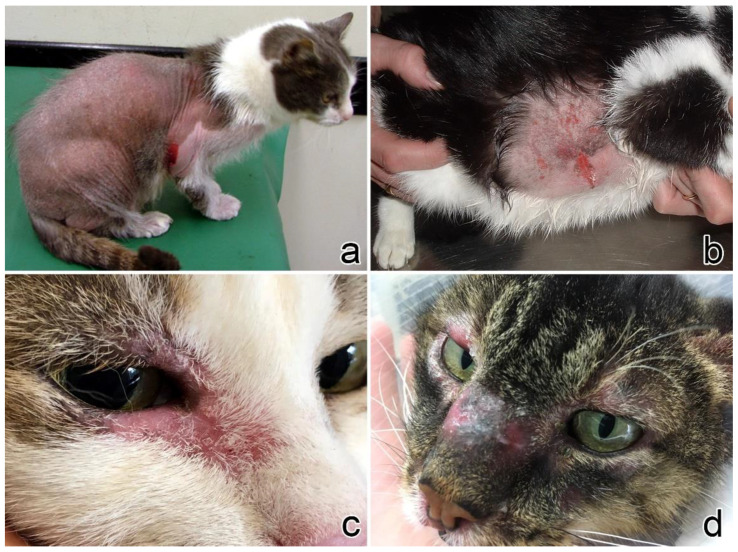
Clinical signs in cats. (**a**) Case no. 3: generalized alopecia and scales (courtesy Dr. Fabrizio Fabbrini). (**b**) Case no. 4: focal alopecia with multifocal ulcers on the abdomen (courtesy Dr. Alessandro Corona). (**c**) Case no. 3: erythema, alopecia, and scales on both right eyelids (courtesy Dr. Sivia Colombo). (**d**) Case no. 14: alopecia, erythema, and swelling of the nose and eyelids (courtesy of Dr. Ilaria Mannara).

In cats, lesions were more frequently focal (n = 9, 47%) or multifocal (n = 7, 37%), whereas the diffuse form was rarely encountered (n = 3, 16%). Head and/or neck were the most affected sites (n = 12, 63%), followed by limbs (n = 7, 37%), thorax and dorsum (n = 5, 26%), axillae and abdomen (n = 5, 26%), flank (n = 4, 21%), tail (n = 1, 5%), and perineum (n = 1, 5%). Alopecia was the most common clinical sign (n = 15, 79%), followed by erythema (n = 12, 63%). Eight (42%) cats presented with scales, and 7 of them (88%) had multifocal or diffuse presentation of the disease (Figure 1).

**Figure 2:**
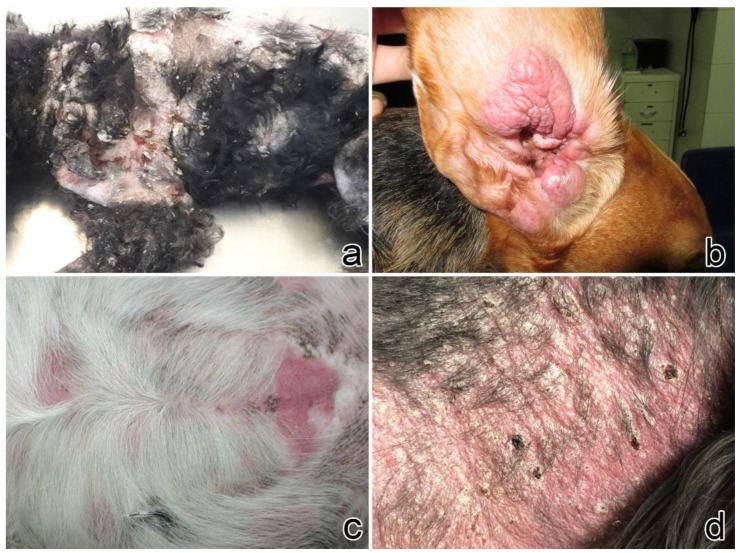
Clinical signs in dogs. (**a**) Case no. 4: severe generalized alopecia with erythematous and exfoliative dermatitis in a Poodle (courtesy Dr. Roberta Gamba). (**b**) Case no. 2: erythematous plaques on the inner surface of the pinna (courtesy Dr. Claudia d’Angeli). (**c**) Case no. 1: multifocal erythematous irregular plaques on the abdomen (courtesy Dr. Francesco Carrani). (**d**) Case no. 6: alopecia, erythema, small yellowish scales on the neck (courtesy Dr. Francesca Carraro).

In dogs, lesions were more frequently multifocal (n = 7, 70%), and rarely focal (n = 2, 20%) or diffuse (n = 1, 10%). The trunk was the most affected site (n = 5, 50%), followed by limbs (n = 4, 40%), abdomen (n = 3, 30%), flank (n = 3, 30%), perianal (n = 2, 20%) and neck, axilla, and ear, each in one dog. Similar to cats, alopecia was the principal clinical sign (n = 7, 70%), followed by erythema (n = 6, 60%). Five (50%) dogs presented with scales, and all of them had a multifocal presentation of the disease (Figure 2).

### 3.2. Histopathology

Detailed results both for cats and dogs are reported in Supplementary Tables S2 and S3. Histograms illustrating the frequency of the lesions are shown in Supplementary Figure S1.

**Figure 3:**
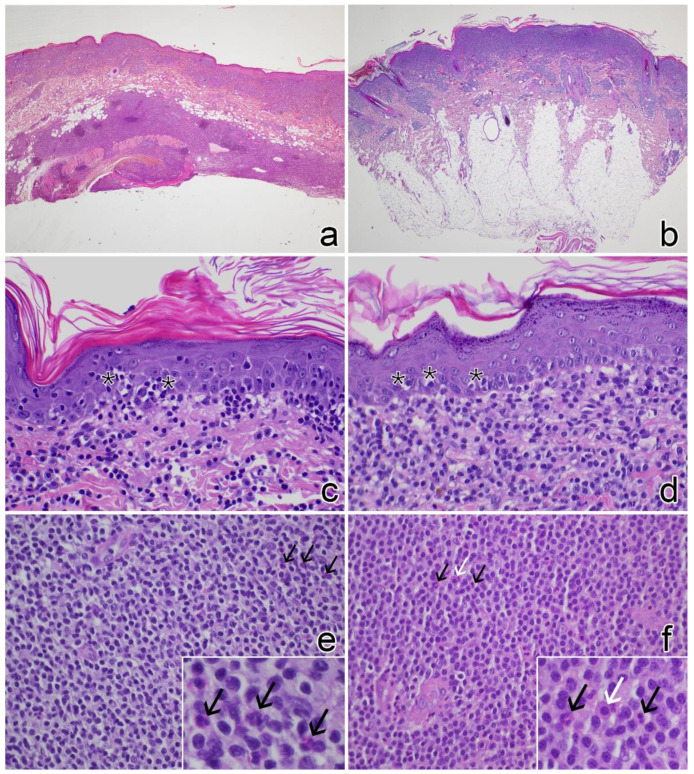
Histological findings in cases of feline and canine cutaneous lymphocytosis (H&E stain). (**a**) Cat no. 3: diffuse lymphocytic infiltrates in the superficial dermis and panniculus, note lymphocyte aggregates in the panniculus (20×). (**b**) Dog no. 6: diffuse lymphocytic infiltrate in the superficial dermis (20×). (**c**) Cat no. 8: mild epitheliotropism by small lymphocyte aggregates (asterisks) (400×). (**d**) Dog no. 4: mild epitheliotropism by single lymphocytes (asterisks) (400×). (**e**) Cat no. 3: diffuse infiltrate of small lymphocytes with few infiltrating eosinophils (black arrows in the figure and in the inset) (400×). (**f**) Cat no. 7: diffuse infiltrate of small lymphocytes with few infiltrating mast cells (white arrow in the figure and in the inset) and eosinophils (black arrows in the figure and in the inset) (400×).

In cats, the most frequent epidermal lesions were mild hyperplasia (n = 19, 100%) with basket-weave hyperkeratosis (n = 11, 58%), epitheliotropism characterized by very few and mostly isolated lymphocytes (n = 10, 53%), and overlying serous-neutrophilic crusts (n = 9, 46%). Epitheliotropism of adnexal structures was mainly characterized by few lymphocytes within the follicular walls (n = 9, 53%); conversely, syringotropism was rare and sebaceous involvement was never detected. Frequently, cats (n = 13, 68%) showed a perivascular to diffuse lymphocyte infiltrate with a full-thickness biopsy distribution. Nodular aggregates of lymphocytes were seen in 7 (37%) cats and composed by small-sized lymphocytes, whereas in 12 (63%) cats the infiltrate was also intermingled with medium-sized lymphocytes. Most lymphocytes were cleaved (n = 14, 74%) and mitoses were rarely observed (n = 3, 16%). Only one cat (case no. 15) showed a mitotic count of 26. Detection of individual mast cells and occasional eosinophils was a frequent finding (n = 12, 63%) (Figure 3a,c,e).

In dogs, the main epidermal lesions were mild hyperplasia (n = 9, 90%) with basket-weave hyperkeratosis (n = 9, 90%) and epitheliotropism characterized by few individual or grouped lymphocytes (n = 7, 70%). Adnexal structure epitheliotropism was rare and mild, either within sweat glands (n = 3, 30%) or follicle wall (n = 2, 20%). Perivascular to diffuse lymphocytes were seen mainly throughout the superficial and mid dermis. The deep dermis/panniculus was less frequently involved and with lower severity compared to cats (n = 5, 50%). Nodular lymphoid aggregates were observed in 6 (60%) dogs. In all cases the infiltrate was composed of small-sized lymphocytes and frequently intermingled with medium-sized indented lymphocytes (n = 8, 80%). Mitoses were observed in 4 (40%) cases, ranging from 1 to 2 per 10 consecutive HPF. Eosinophils and mast cells were commonly observed within the infiltrate (Figure 3b,d,f).

### 3.3. Immunophenotyping

**Figure 4:**
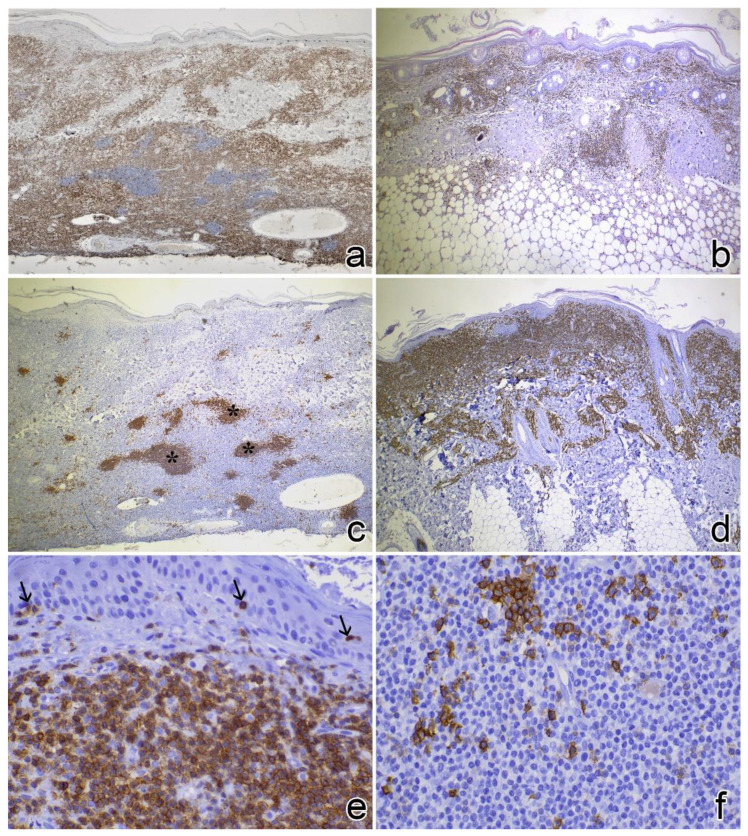
Immunohistochemical findings of feline and canine lymphocytosis (peroxidase method). (**a**) Cat no. 3: infiltrating cells are diffusely positive with the anti-CD3 antibody (40×). (**b**) Dog no. 8: infiltrating cells are diffusely positive with the anti-CD3 antibody (40×). (**c**) Cat no. 3: deep nodular aggregates of lymphocytes (asterisks) are positive for the anti-CD20 antibody (B lymphocytes) (40×). (**d**) Dog no. 6: infiltrating cells are diffusely positive with the anti-CD20 antibody for B lymphocytes (40×). (**e**) Cat no. 6: lymphocytes are diffusely positive for anti-CD3 antibody, note the scant epitheliotropism (black arrows, 400×). (**f**) Dog no. 3: CD3+ T lymphocytes are present, single or in small aggregates (400×).

Immunohistochemical results are reported in Supplementary Tables S2 and S3. All feline CLs (n = 19, 100%) stained positive for CD3 (score 3). A mild CD20+ lymphocytic infiltrate (score 1) was detected in one cat only. Sixteen (84%) cats showed nodular aggregates of CD20+ lymphocytes within the T-cell infiltrate, more frequently in the deep portion of the skin. CD3+ nodular aggregates were found in 2 cases (11%) (Figure 4a,c,e).

In dogs, CD3+ lymphocytes were observed in 9 (90%) cases (78% scored 3). In 5 (50%) dogs, CD20+ lymphocytic infiltrates were also observed. Lymphocytes were negative both for CD3 and CD20 in one (10%) case. By immunohistochemistry nodular aggregates of lymphocytes were finally detected in all cases and predominantly composed of CD20+ lymphocytes in 7 (70%) dogs and CD3+ lymphocytes in 3 (30%) dogs (Figure 4b,d,f).

### 3.4. Clonality Test

No clonal rearrangements were identified in feline CL (Table 3). Conversely, a clonal BCR rearrangement was found in 4 (40%) dogs, and a clonal TCR rearrangement in 1 (10%) animal. To note, one dog showed rearrangements both in the BCR and the TCR gene loci (Table 4).

### 3.5. Staging and Clinical Follow-Up

Complete follow-up data are shown in Table 3 and Table 4.

Among cats, 8 (58%) underwent staging, and only one presented submandibular nodal involvement. The other 7 cats had negative staging. Information regarding treatment was retrieved for 18 cases: 8 (44%) cats received steroids alone or in combination with cyclosporine, 4 (22%) cats received oral chlorambucil with or without prednisolone, 2 (11%) cats underwent surgical removal of the single cutaneous plaque/nodule, whereas 4 (22%) cats received no treatment. Among cats receiving steroids only, 4 obtained a partial remission, 1 a complete remission, whereas 1 cat was stable. Among cats receiving chemotherapy, 3 were stable and 1 obtained a partial remission. All cats receiving no treatment had stable disease. The two operated cats did not progress. Follow-up data were available for 17 cats: at data analysis closure, 8 cats were alive after a median follow-up of 570 days (range 81–1680), 7 were dead (3 for unrelated causes and 4 for unknown causes), and 3 were lost to follow-up. Median OS was 1080 days (range: 30–1680).

**Figure 5:**
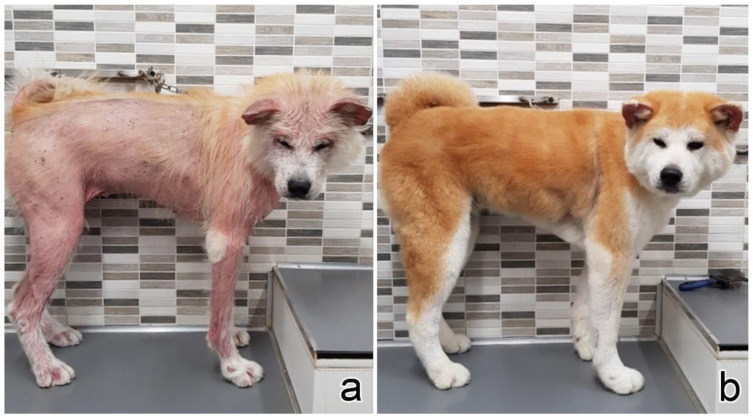
Clinical follow up of dog no. 9. (**a**) The dog at presentation: severe generalized erythematous and exfoliative dermatitis. (**b**) The dog at 11-month follow-up after removal of all drugs: relevant improvement of clinical signs with almost complete hair regrowth (courtesy Dr. Roberto Damiani).

Among dogs, 8 (80%) underwent staging: One dog had peripheral nodal involvement and one had a concomitant chronic lymphocytic leukaemia. The other 6 dogs had negative staging. Five (50%) dogs received chemotherapy (lomustine, n = 1; chlorambucil, n = 1, or both as monotherapy, n = 3) and steroids, 1 (10%) was treated with steroids and cyclosporine, 3 (30%) underwent surgical excision of the cutaneous lesions, and 1 (10%) dog received no treatment. Among dogs receiving chemotherapy, 2 had a partial response, 1 was stable, and 1 progressed over time. Another dog receiving chemotherapy showed a stable partial response when therapy was suspended (case no. 9). In detail, this dog started treatment with steroids followed by cyclosporine, chlorambucil, and lomustine, the latter given in two administrations, without showing any improvement (stable disease). Six weeks after discontinuation of lomustine remission of cutaneous lesions started, the dog then showed complete hair regrowth although erythema and scales were still present at the time of writing (7 months) (Figure 5). The dog receiving steroids and cyclosporine had stable disease. The dog receiving no treatment had a spontaneous complete remission of the cutaneous lesions. Three dogs that were operated obtained a complete remission. Follow-up data were available for 9 dogs: At data analysis closure, 6 dogs were alive after a median follow-up of 240 days (range 150–720), 2 were lost to follow-up, and 2 were dead (one for progressive disease and one for unknown causes). Median OS was not reached.

Among the 6 dogs with clonal rearrangements, 2 achieved complete remission after surgical resection of the lesion, 2 achieved partial remission, and 2 dogs remained stable after treatment with corticosteroids and cyclosporine or corticosteroids and chemotherapy, respectively. Interestingly, one dog (case no. 9) with clonal TCR rearrangement and receiving different therapy regimens without any improvement (see above) showed a stable partial remission after drug withdrawal. Five out of 6 dogs with clonal rearrangements were alive at data analysis closure, with a median follow-up of 240 days. One dog was lost to follow-up.

## 4. Discussion

We describe here a case series of 19 cats and 10 dogs diagnosed with CL according to previously established histopathological criteria [4,5,7]. A number of histological scores were considered to highlight similarities between the two species and identify differences between CL and CEL.

In cats, CL mainly affected old female domestic short hair breeds. Alopecia and erythema were the main clinical signs when focal lesions were present, whereas scales were frequently observed in multifocal or diffuse disease presentation. These results are in accordance with previous studies [4,6,7,18], but considering the high heterogeneity of the lesions and the clinical response to treatment several differential diagnoses should be always considered when diagnosing CL clinically. Therefore, skin biopsies should be always included in the diagnostic workup of the disease in combination with a complete dermatological screening. Thus, inclusion criteria were very strict in our case series, indeed only animals with a biopsy with histopathological features compatible with CL were considered. The selection was irrespective of the clinical history and presentation.

The lesions extended from the superficial to the deep dermis and superficial panniculus. Lymphocytes were diffusely small and well-differentiated, compatible with a mature stage of development. Histologically, a form of CEL was always considered as first differential diagnosis [18,19], but here we identified morphological features differentiating the two diseases, including the degree of epitheliotropism, B-cells distribution, and the presence of a variety of inflammatory cells. Previously, Fontaine et al. reported evidence of epitheliotropism in feline CEL [20] and neoplastic cells formed small aggregates, Pautrier micro-abscesses, or had a panepidermal localization. In our case series, epitheliotropism, defined as focal infiltration of few individual small and well-differentiated lymphocytes, was only observed in half of the cases. These results suggest that the absence or a scant epitheliotropism in association with a diffuse cutaneous infiltration of small well-differentiated lymphocytes may represent an important diagnostic feature to differentiate between CL and CEL. Plasma cells and macrophages were the most represented inflammatory cells in feline CEL [20]. Conversely, they were rare in CL, but eosinophils and mast cells were frequently found (13 cases, 68%). Furthermore, the number of mast cells might have been further underestimated since toluidine blue staining was not performed here.

Regarding the phenotype, lymphocytic infiltrates were diffusely positive for CD3, and although in accordance with the published literature, the relevance of this data remains limited since CEL in cats are reported originating more frequently from T-cells, limiting the diagnostic significance of this antibody in discriminating the two conditions [4,6]. However, a total of 16 cats (84%) had CD20+ nodular aggregates in the dermis. Among them, 7 cats showed a follicular differentiation by H&E stain (37%), but polarization and mantle zones were always absent (data not shown). Literature reports a sparse and rare infiltration of B-cells in cats with T-cell CEL and most commonly as dermal nodular aggregates [20]. The distribution of B-cells in CL needs further investigations and a higher number of cases. A further confirmation that our feline CL cases were reactive disorder rather than neoplastic in origin was the absence of clonal rearrangements in all cases. This result is discordant in comparison with the study of Gilbert et al. [4], where 72% of cats with CL showed a TCR clonal rearrangement. Unfortunately, comparisons are not possible since technical description of PARR assay, including primers and protocols, was not reported.

To the authors’ knowledge, standard treatments for feline CL are not described. Here, cats were treated with a variety of protocols or even not treated at all and showed a long-term survival with stable disease or progression and eventually died for unrelated or unknown causes when not treated. This finding is again in contrast with survival data reported by Fontaine et al. [20] for feline CEL that was dramatically short, ranging from 1 to 6 months. On the other side, the long survival obtained in our study reinforces the diagnosis of CL compared to CEL, known to be aggressive and lethal.

Unfortunately, only a reduced number of dogs met the inclusion criteria, confirming the lower prevalence of CL in this species compared to cats. Many breeds were represented in contrast with previously published data [5]. Old dogs were mainly affected, and females were overrepresented. Most of the dogs showed multifocal to diffuse lesions, and CEL was always included as differential diagnosis. Histological findings were very similar to those described in cats, but some differences were identified, including a high degree of hyperkeratosis, a lower lymphocytic infiltration of the deep dermis and panniculus, and a more frequent epitheliotropism. Two out of 3 cases with moderate epitheliotropism had a polyclonal rearrangement, ruling out the diagnosis of epitheliotropic lymphoma. Finally, nodular lymphocyte aggregates were similarly found.

However, major differences between the two species were highlighted when examining immunohistochemistry and PARR results. Indeed, a mixed diffuse infiltration of T- and B-cells was observed in 3 dogs, whereas one dog showed only CD20+ lymphocytes. Lymphocytosis characterized by B cell diffuse infiltrates has been described in humans [21], but not in cats and again confirmed by our results. Finally, in one dog the infiltrate was negative for both CD3 and CD20.

Differently from feline CL, monoclonal rearrangements were detected in 6 dogs, however no morphological and clinical differences were observed compared to dogs characterized by polyclonal rearrangements. Further, none of the dogs with PARR clonality died from the disease despite a long-term follow-up. The diagnostic relevance of a clonal PARR result for skin lesions remains still undetermined in veterinary medicine. Given the reported sensitivity and specificity of the technique, a possible explanation may suggest a false positive result [14]. Another intriguing hypothesis implies the presence of a pre-neoplastic lesion eventually evolving into overt lymphoma.

Many different treatment protocols were adopted here, and a uniform response trend was not obtained, also altered by the inclusion of both PARR-positive and negative cases. Interestingly, a complete remission was spontaneously obtained in one untreated dog and a long and stable partial remission was achieved even after treatment interruption in another dog. For this dog a beneficial late effect of lomustine cannot be excluded and since the reported median survival time of lomustine for treatment of canine CEL is 6 months [22], a longer follow-up time is needed for any conclusion. Four out of 5 dogs that were treated with chemotherapy presented either positive staging or concomitant haematological abnormalities; however, the treatment response was overall unsatisfying. Based on these findings, the biological behavior of canine CL remains unpredictable.

## 5. Conclusions

**Figure 6:**
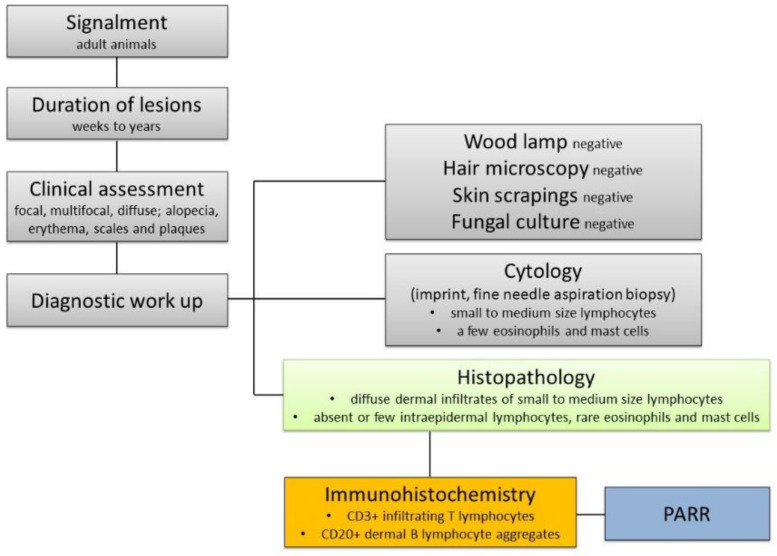
Suggested clinical and diagnostic algorithm in suspected cases of cutaneous lymphocytosis.

In conclusion, we describe here a series of feline and canine CL, highlighting similarities and differences between the two species, and propose a clinical decision-making flow chart in suspected cases of CL (Figure 6). Our results suggest that the discrimination between CL and CEL is more challenging in dogs than in cats. Differently from what previously reported, our data support that a reactive nature of CL is more likely, at least in cats. Prospective studies are needed including a higher number of cases, with standardized diagnostic and staging workup and treatment regimen, in order to investigate the diagnostic and prognostic features of CL in both species.

## Figures and Tables

**Table 1:** Signalment and clinical findings in cats with cutaneous lymphocytosis.

Case No.	Signalment			Clinical Features of the Lesions											Other Clinical Findings
	Breed	Sex	Age (Years)	Site	Longest Diameter (cm)	Distribution	Duration (Months)	U	Pr	A	Er	Ex	Pl	Additional Features	
1	DSH	F	12	Head, neck, forelimb (shoulder)	1.5	Multifocal	3						X	Nodules	None
2	DSH	M	15	Head (upper lip)	4	Focal	6			X	X		X		None
3	DSH	M	14	Neck, dorsum, perineum, limbs		Diffuse	24	X	X	X		X		Papules, crusts	Otitis
4	DSH	F	14	Flank	4	Focal	2 weeks	X	X	X		X			None
5	DSH	M	9	Head (upper lip, periocular)	1	Focal	18	X	X	X	X				None
6	DSH	M	11	Head (eyelid)		Focal	12			X					
7	DSH	M	16	Thorax-lumbar	10	Focal	24	X		X	X		X		None
8	DSH	F	10	Neck, axillae, dorsum		Multifocal	7		X	X	X	X			Anorexia, hyperthermia, melena, weight loss
9	DSH	F	14	Head (periocular), hindlimb (metatarsus), tail	5	Multifocal	6			X	X	X	X		None
10	DSH	F	10	Head (upper lip), flank (bilateral)	5	Multifocal	3			X	X		X	Hyperpigmentation	None
11	DSH	F	10	Flank		Focal	9	X							
12	DSH	F	13	Forelimb (elbow)	4	Focal	2			X	X		X		None
13	DSH	F	10	Thorax, axillae	4	Multifocal	12			X	X	X			None
14	DSH	M	7	Head, abdomen (bilateral)		Diffuse	3		X	X	X	X			Weight loss, submandibular lymphadenomegaly, FIV-positive
15	DSH	F	9	Abdomen	1	Focal	2							Nodule	None
16	DSH	M	14	Hindlimb (3rd digit)	0.2	Focal	12	X		X				Nodule	None
17	DSH	F	7	Head (perilabial), neck, abdomen, trunk, hindlimb (thighs)		Diffuse	24	X		X	X	X			Bronchitis
18	DSH	F	13	Neck, flank, hindlimb (thigh)	20	Multifocal	60		X		X	X			None
19	DSH	F	14	Head, dorsum, abdomen	30	Multifocal	6	X	X	X	X			Dryness, crusts	None

A = alopecia; DSH = domestic short hair; Er = erythema; Ex = exfoliation; FIV = feline immunodeficiency virus; Pl = plaques; Pr = pruritus; U = ulcer.

**Table 2:** Signalment and clinical findings in dogs with cutaneous lymphocytosis.

Case No.	Signalment			Clinical Features of the Lesions											Other Clinical Findings
	Breed	Sex	Age (Years)	Site	Longest Diameter (cm)	Distribution	Duration (Months)	U	Pr	A	Er	Ex	Pl	Additional Features	
1	Beagle	F	13	Abdomen, hindlimb	2	Multifocal	2 weeks				X				None
2	Beagle	M	6	Head (ear), perianal	3	Multifocal	1			X	X		X		None
3	Shar Pei	F	12	Thorax	2	Multifocal	1			X		X			None
4	Toy poodle	F	13	Dorsum, flank, abdomen	2	Multifocal	1	X		X	X	X			Lymphadenomegaly
5	Mixed breed	M	8	Thorax	7	Focal	5						X		None
6	Breton	M	11	Neck, flank, thorax	20	Multifocal	2			X	X	X		Crusts	None
7	Mixed breed	F	15	Perianal	8	Focal	1	X		X			X		Lymphocytic leukemia, mesenchymal gastric tumour
8	Mixed breed	F	12	Trunk, flank	2	Multifocal	10	X	X		X	X	X		Ataxia, hypoglycemic crisis
9	Akita Inu	F	9	Axilla, forelimb (shoulder), hindlimb		Diffuse	12		X	X	X				Anemia, thrombocytopenia
10	Dachshund	F	8	Abdomen	5	Multifocal	2 weeks			X		X	X		None

A = alopecia; Er = erythema; Ex = exfoliation; Pl = plaques; Pr = pruritus; U = ulcer.

**Table 3:** Data on clonality testing (PARR), staging, therapy, response to therapy, overall survival, and cause of death in cats with cutaneous lymphocytosis.

Case No.	PARR	Staging	Therapy	Response to Therapy	Overall Survival (Days)	Cause of Death
1	neg	neg (HB, U, Rx)	Corticosteroids	PR	365	Lost to follow-up
2	neg	neg (HB)	None	SD	1080	Unrelated (renal failure)
3	neg	n.a.	None	SD	720	Unrelated (renal failure)
4	neg	n.a.	None	SD	240	Unknown
5	neg	n.a.	Corticosteroids, chlorambucil	PR	540	Lost to follow-up
6	neg	n.a.	None	SD	90	Unrelated (renal failure)
7	neg	n.a.	Corticosteroids, chlorambucil	SD	570	Lost to follow-up
8	neg	n.a.	Corticosteroids	SD	30	Unknown
9	neg	neg (lnC)	Corticosteroids, cyclosporine	PR	480	Alive
10	neg	neg (HB, U)	Corticosteroids, cyclosporine	PR	1080	Alive
11	neg	n.a.	n.a.	n.a.	n.a.	n.a.
12	neg	n.a.	Corticosteroids	CR	1080	Alive
13	neg	n.a.	Chlorambucil	SD	81	Alive
14	neg	Submandibular lymphoadenomegaly (U, CT)	Corticosteroids	PR	90	Unknown
15	neg	n.a.	Surgery	CR	365	Unknown (not investigated visceral mass)
16	neg	n.a.	Surgery	CR	570	Alive
17	neg	neg (HB, U)	Corticosteroids	PR	180	Alive
18	neg	neg (HB, spC, livC)	Corticosteroids	PR	1680	Alive
19	neg	neg (HB, spC, livC)	Corticosteroids, chlorambucil	SD	1080	Alive

CR = complete remission; HB = hemato-biochemistry; livC = liver cytology; lnC = lymph node cytology; n.a. = not available; neg = negative; PR = partial remission; Rx = radiography; SD = stable disease spC = spleen cytology; CT = computed tomography; U = urinary assay.

**Table 4:** Data on clonality testing (PARR), staging, therapy, response to therapy, overall survival, and cause of death in dogs with cutaneous lymphocytosis.

Case No.	PARR	Staging	Therapy	Response to Therapy	Overall Survival (Days)	Cause of Death
1	neg	n.a.	None	CR	28	Lost to follow-up
2	TCR/BCR clonal	neg (lnC)	Corticosteroids, cyclosporine	SD	365	Lost to follow-up
3	BCR clonal	neg (HB, US, spC, livC)	Surgery	CR	630	Alive
4	neg	neg (HB, US, spC, livC)	Lomustine, chlorambucil *	PD	360	Related (euthanasia for progressive disease)
5	neg	n.a.	Surgery	CR	720	Alive
6	BCR clonal	neg (HB, US, spC, livC)	Corticosteroids, chlorambucil	SD	240	Alive
7	BCR clonal	lymphocitic leukemia (HB, U)	Corticosteroids, lomustine	PR	240	Alive
8	neg	neg (HB, US, spC, livC)	Lomustine, chlorambucil *	PR	n.a.	Unknown
9	TCR clonal	mild anemia and thrombocytopenia (HB)	Corticosteroids, cyclosporine, chlorambucil, lomustine *	PR (after drug withdrawal)	334	Alive
10	BCR clonal	neg (U, lnC)	Surgery	CR	150	Alive

BCR = B-cell receptor; CR = complete remission; HB = hemato-biochemistry; livC = liver cytology; lnC = lymph node cytology; n.a. = not available; neg = negative; PD = progressive disease; PR = partial remission; SD = stable disease; spC = spleen cytology; TCR = T-cell receptor; U = urinary assay; US = ultrasound; * = monotherapy (not in combination).

## Data Availability

Not applicable.

## Supplementary Materials

The following supporting information can be downloaded at: https://www.mdpi.com/article/10.3390/vetsci9010026/s1, Table S1: Primer nucleotide sequences for PARR assay; Table S2: Score assessment of histological and immunohistochemical findings in cases of feline lymphocytosis; Table S3: Score assessment of histological and immunohistochemical findings in cases of canine lymphocytosis; Figure S1: Frequency of the histological and immunohistochemical findings in feline and canine cutaneous lymphocytosis.
