# Supplementary material for: Feline and Canine Cutaneous Lymphocytosis: Reactive Process or Indolent Neoplastic Disease?
Source: Vet Sci. 2022 Jan 11;9(1):26. doi: 10.3390/vetsci9010026 (PMC8778986; doi:10.3390/vetsci9010026)
Supplement: Supplementary file 1 [file vetsci-09-00026-s001.zip › Supplementary Figure S1.pptx]

## Slide 1
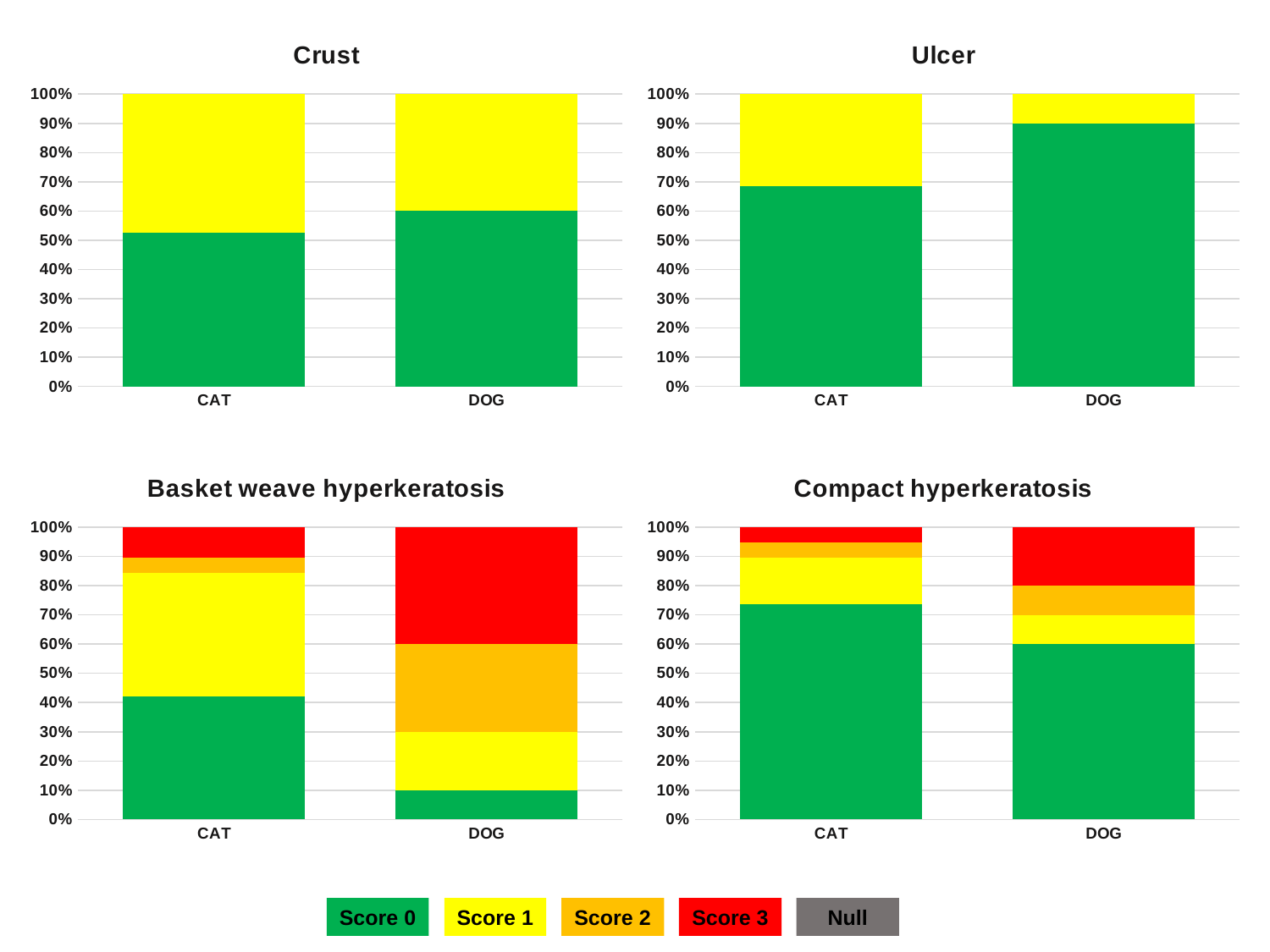

### Chart: Ulcer
| Category | 0 | 1 | 2 | 3 | nd |
|---|---|---|---|---|---|
| CAT | 13.0 | 6.0 | None | None | None |
| DOG | 9.0 | 1.0 | None | None | None |
### Chart: Crust
| Category | 0 | 1 | 2 | 3 | nd |
|---|---|---|---|---|---|
| CAT | 10.0 | 9.0 | None | None | None |
| DOG | 6.0 | 4.0 | None | None | None |
### Chart: Basket weave hyperkeratosis
| Category | 0 | 1 | 2 | 3 | nd |
|---|---|---|---|---|---|
| CAT | 8.0 | 8.0 | 1.0 | 2.0 | None |
| DOG | 1.0 | 2.0 | 3.0 | 4.0 | None |
### Chart: Compact hyperkeratosis
| Category | 0 | 1 | 2 | 3 | nd |
|---|---|---|---|---|---|
| CAT | 14.0 | 3.0 | 1.0 | 1.0 | None |
| DOG | 6.0 | 1.0 | 1.0 | 2.0 | None |Null
Score 2
Score 3
Score 0
Score 1

## Slide 2
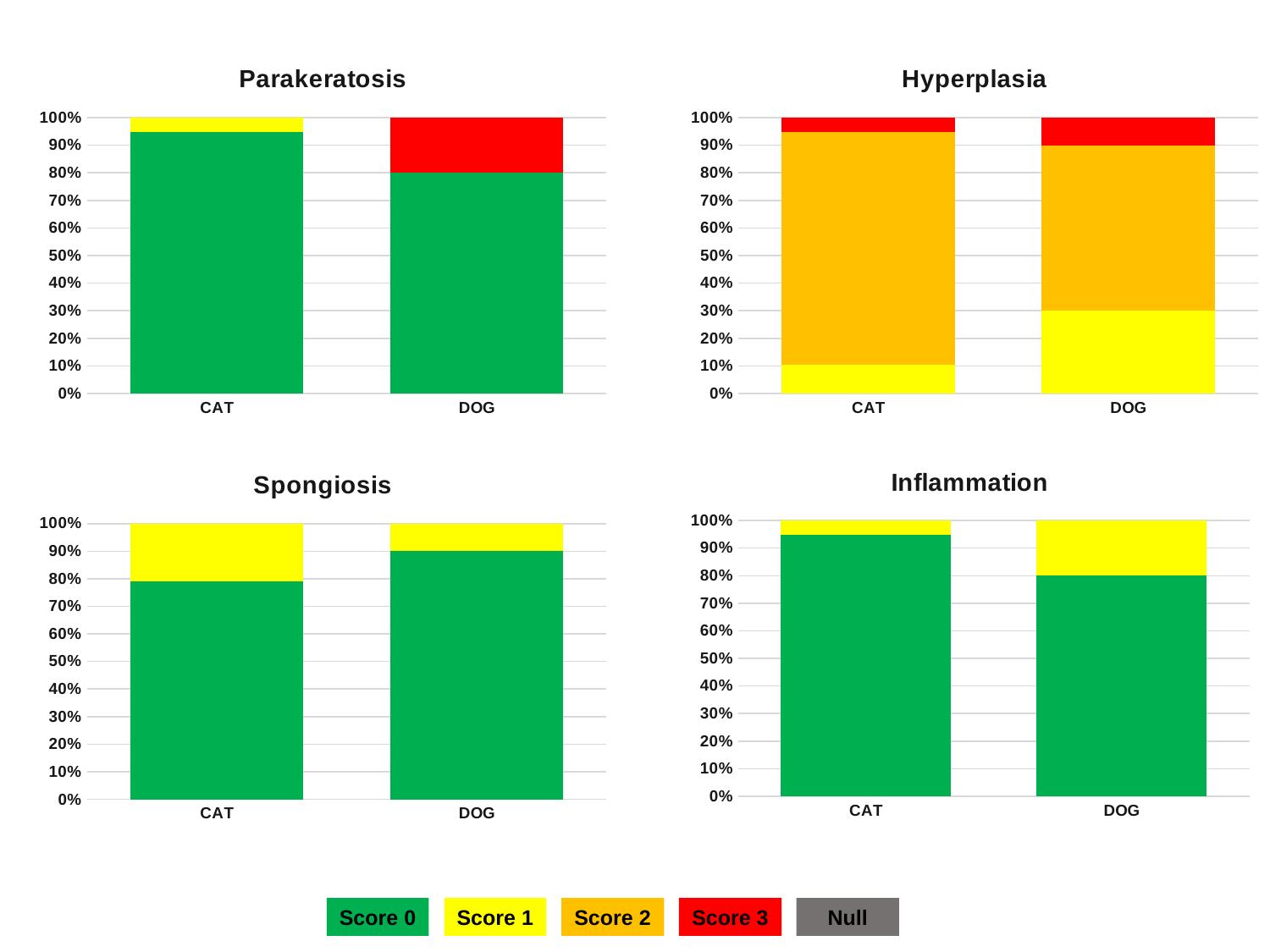

### Chart: Parakeratosis
| Category | 0 | 1 | 2 | 3 | nd |
|---|---|---|---|---|---|
| CAT | 18.0 | 1.0 | None | None | None |
| DOG | 8.0 | None | None | 2.0 | None |
### Chart: Hyperplasia
| Category | 0 | 1 | 2 | 3 | nd |
|---|---|---|---|---|---|
| CAT | None | 2.0 | 16.0 | 1.0 | None |
| DOG | None | 3.0 | 6.0 | 1.0 | None |
### Chart: Inflammation
| Category | 0 | 1 | 2 | 3 | nd |
|---|---|---|---|---|---|
| CAT | 18.0 | 1.0 | None | None | None |
| DOG | 8.0 | 2.0 | None | None | None |
### Chart: Spongiosis
| Category | 0 | 1 | 2 | 3 | nd |
|---|---|---|---|---|---|
| CAT | 15.0 | 4.0 | None | None | None |
| DOG | 9.0 | 1.0 | None | None | None |Null
Score 2
Score 3
Score 0
Score 1

## Slide 3
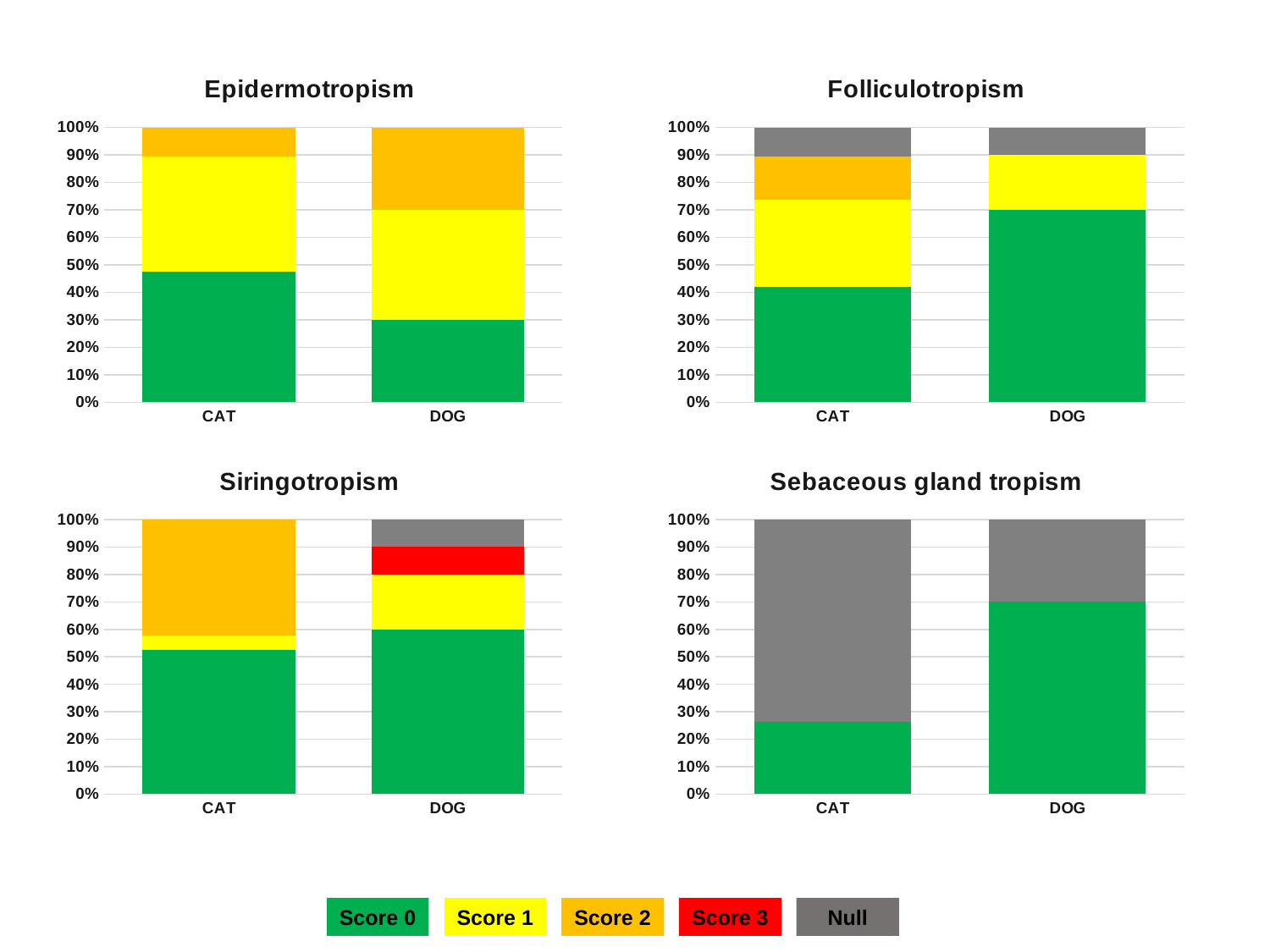

### Chart: Epidermotropism
| Category | 0 | 1 | 2 | 3 | nd |
|---|---|---|---|---|---|
| CAT | 9.0 | 8.0 | 2.0 | None | None |
| DOG | 3.0 | 4.0 | 3.0 | None | None |
### Chart: Folliculotropism
| Category | 0 | 1 | 2 | 3 | nd |
|---|---|---|---|---|---|
| CAT | 8.0 | 6.0 | 3.0 | None | 2.0 |
| DOG | 7.0 | 2.0 | None | None | 1.0 |
### Chart: Sebaceous gland tropism
| Category | 0 | 1 | 2 | 3 | nd |
|---|---|---|---|---|---|
| CAT | 5.0 | None | None | None | 14.0 |
| DOG | 7.0 | None | None | None | 3.0 |
### Chart: Siringotropism
| Category | 0 | 1 | 2 | 3 | nd |
|---|---|---|---|---|---|
| CAT | 10.0 | 1.0 | 8.0 | None | None |
| DOG | 6.0 | 2.0 | None | 1.0 | 1.0 |Null
Score 2
Score 3
Score 0
Score 1

## Slide 4
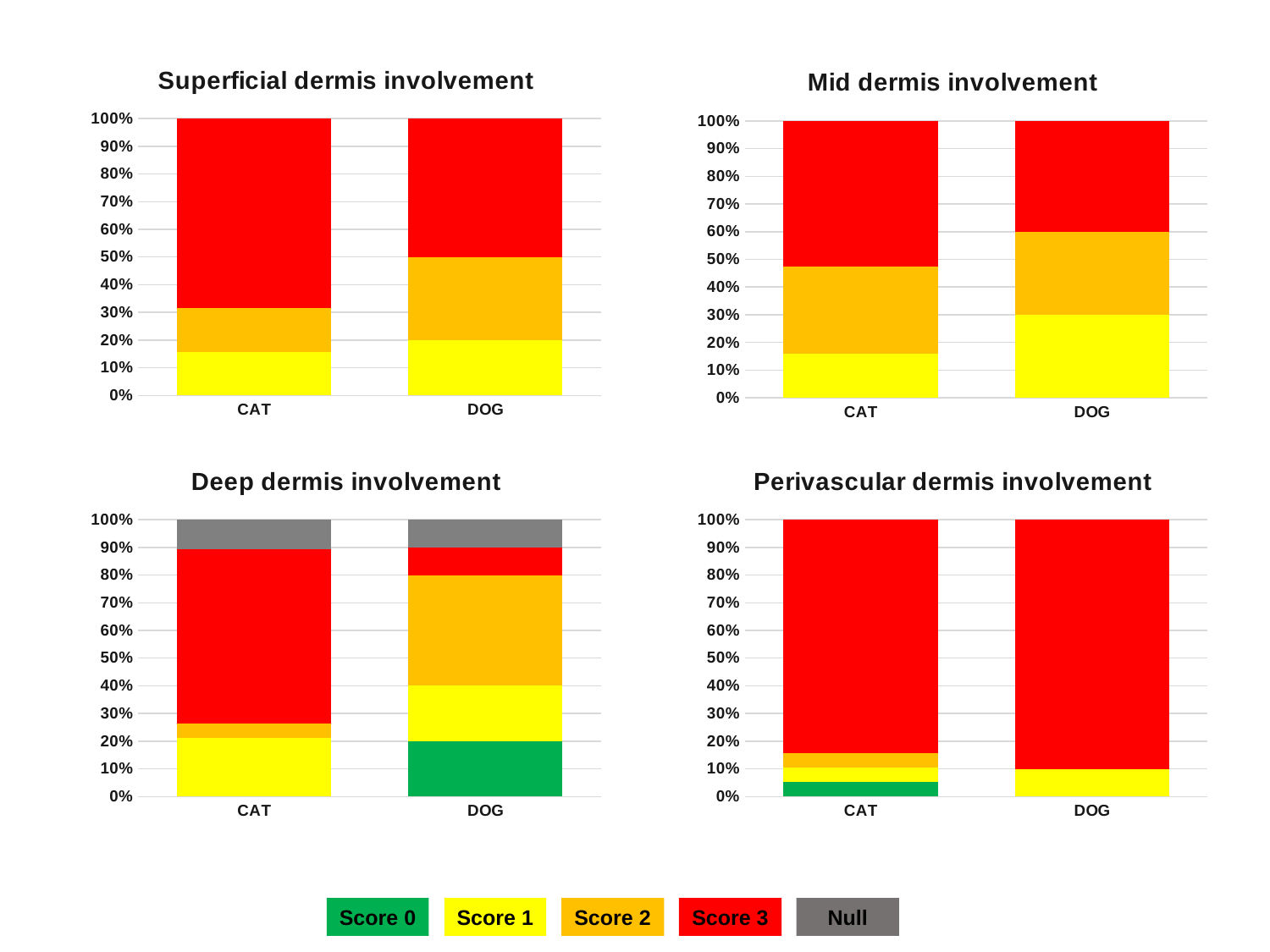

### Chart: Superficial dermis involvement
| Category | 0 | 1 | 2 | 3 | nd |
|---|---|---|---|---|---|
| CAT | None | 3.0 | 3.0 | 13.0 | None |
| DOG | None | 2.0 | 3.0 | 5.0 | None |
### Chart: Mid dermis involvement
| Category | 0 | 1 | 2 | 3 | nd |
|---|---|---|---|---|---|
| CAT | None | 3.0 | 6.0 | 10.0 | None |
| DOG | None | 3.0 | 3.0 | 4.0 | None |
### Chart: Deep dermis involvement
| Category | 0 | 1 | 2 | 3 | nd |
|---|---|---|---|---|---|
| CAT | None | 4.0 | 1.0 | 12.0 | 2.0 |
| DOG | 2.0 | 2.0 | 4.0 | 1.0 | 1.0 |
### Chart: Perivascular dermis involvement
| Category | 0 | 1 | 2 | 3 | nd |
|---|---|---|---|---|---|
| CAT | 1.0 | 1.0 | 1.0 | 16.0 | None |
| DOG | None | 1.0 | None | 9.0 | None |Null
Score 2
Score 3
Score 0
Score 1

## Slide 5
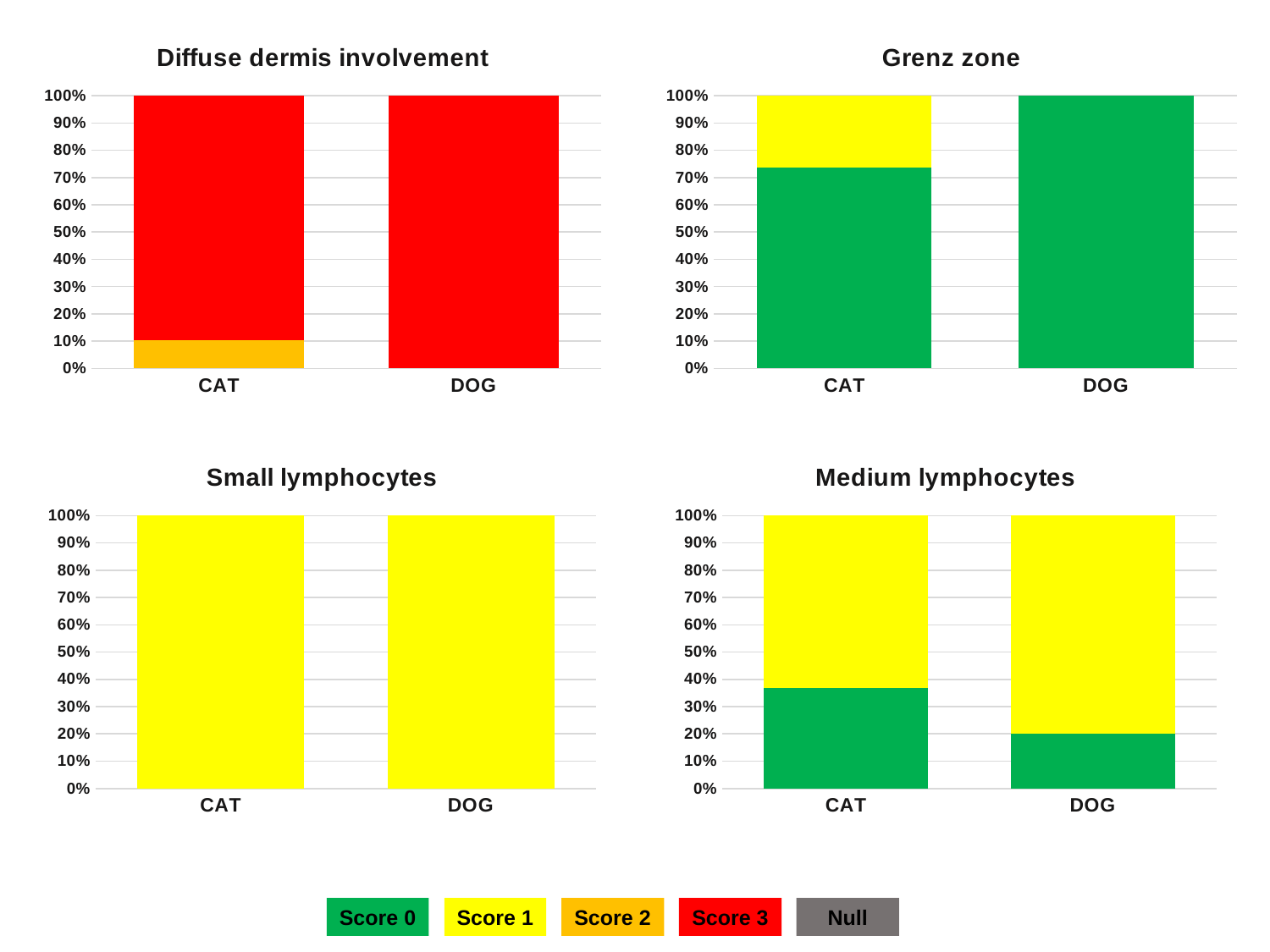

### Chart: Diffuse dermis involvement
| Category | 0 | 1 | 2 | 3 | nd |
|---|---|---|---|---|---|
| CAT | None | None | 2.0 | 17.0 | None |
| DOG | None | None | None | 10.0 | None |
### Chart: Grenz zone
| Category | 0 | 1 | 2 | 3 | nd |
|---|---|---|---|---|---|
| CAT | 14.0 | 5.0 | None | None | None |
| DOG | 10.0 | None | None | None | None |
### Chart: Small lymphocytes
| Category | 0 | 1 | 2 | 3 | nd |
|---|---|---|---|---|---|
| CAT | None | 19.0 | None | None | None |
| DOG | None | 10.0 | None | None | None |
### Chart: Medium lymphocytes
| Category | 0 | 1 | 2 | 3 | nd |
|---|---|---|---|---|---|
| CAT | 7.0 | 12.0 | None | None | None |
| DOG | 2.0 | 8.0 | None | None | None |Null
Score 2
Score 3
Score 0
Score 1

## Slide 6
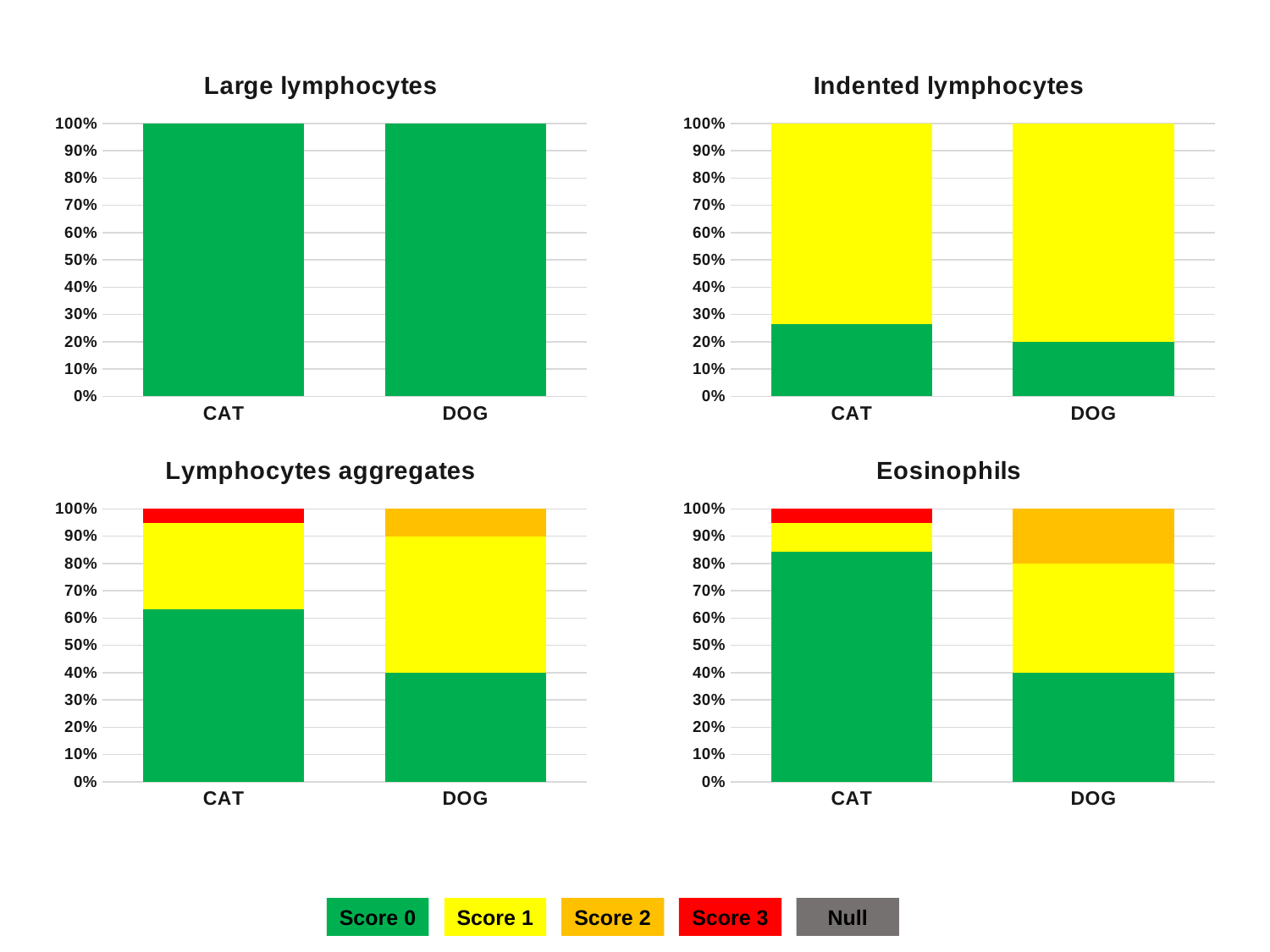

### Chart: Large lymphocytes
| Category | 0 | 1 | 2 | 3 | nd |
|---|---|---|---|---|---|
| CAT | 19.0 | None | None | None | None |
| DOG | 10.0 | None | None | None | None |
### Chart: Indented lymphocytes
| Category | 0 | 1 | 2 | 3 | nd |
|---|---|---|---|---|---|
| CAT | 5.0 | 14.0 | None | None | None |
| DOG | 2.0 | 8.0 | None | None | None |
### Chart: Lymphocytes aggregates
| Category | 0 | 1 | 2 | 3 | nd |
|---|---|---|---|---|---|
| CAT | 12.0 | 6.0 | None | 1.0 | None |
| DOG | 4.0 | 5.0 | 1.0 | None | None |
### Chart: Eosinophils
| Category | 0 | 1 | 2 | 3 | nd |
|---|---|---|---|---|---|
| CAT | 16.0 | 2.0 | None | 1.0 | None |
| DOG | 4.0 | 4.0 | 2.0 | None | None |Null
Score 2
Score 3
Score 0
Score 1

## Slide 7
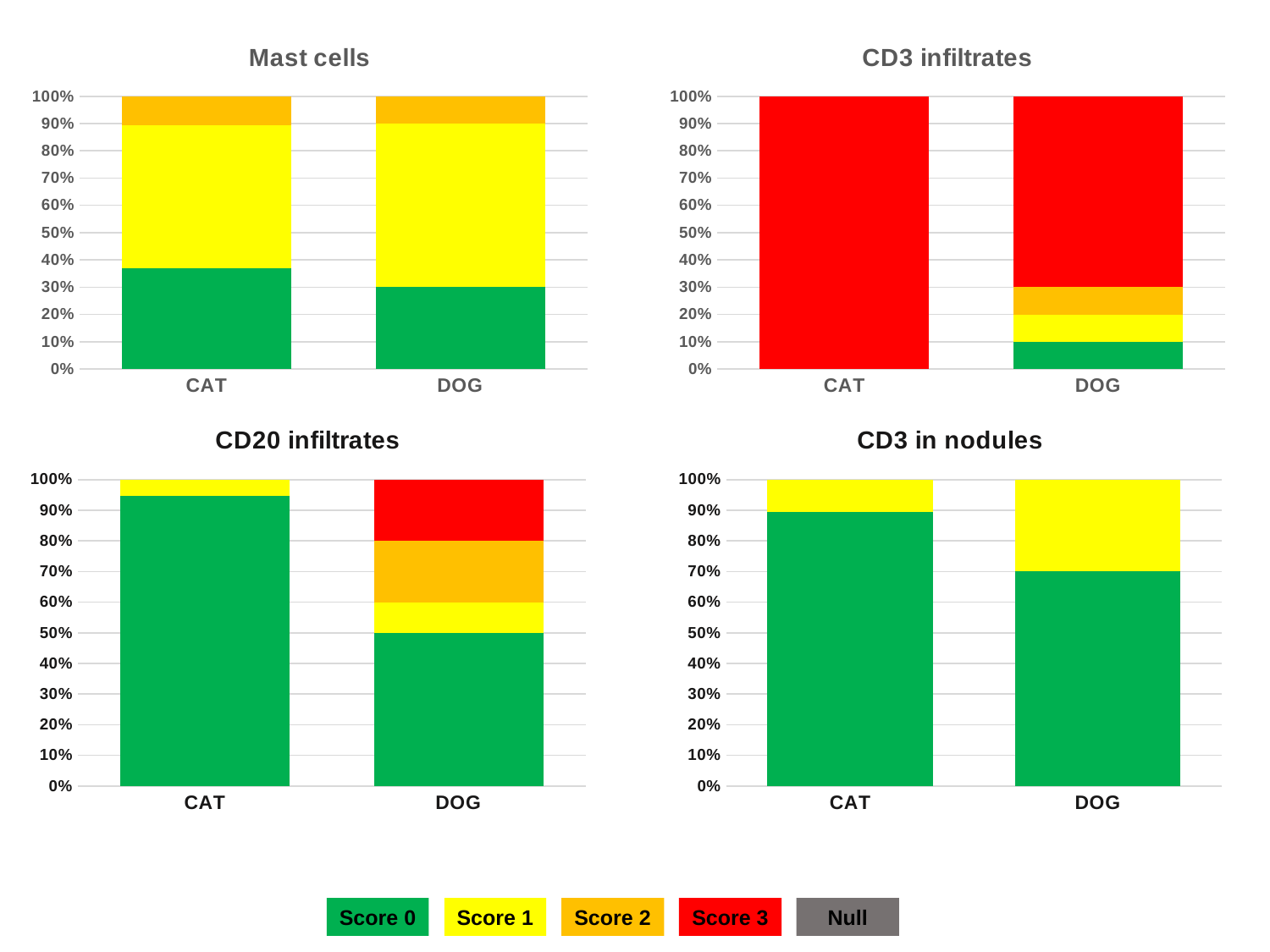

### Chart: Mast cells
| Category | 0 | 1 | 2 | 3 | nd |
|---|---|---|---|---|---|
| CAT | 7.0 | 10.0 | 2.0 | None | None |
| DOG | 3.0 | 6.0 | 1.0 | None | None |
### Chart: CD3 infiltrates
| Category | 0 | 1 | 2 | 3 | nd |
|---|---|---|---|---|---|
| CAT | None | None | None | 19.0 | None |
| DOG | 1.0 | 1.0 | 1.0 | 7.0 | None |
### Chart: CD20 infiltrates
| Category | 0 | 1 | 2 | 3 | nd |
|---|---|---|---|---|---|
| CAT | 18.0 | 1.0 | None | None | None |
| DOG | 5.0 | 1.0 | 2.0 | 2.0 | None |
### Chart: CD3 in nodules
| Category | 0 | 1 | 2 | 3 | nd |
|---|---|---|---|---|---|
| CAT | 17.0 | 2.0 | None | None | None |
| DOG | 7.0 | 3.0 | None | None | None |Null
Score 2
Score 3
Score 0
Score 1

## Slide 8
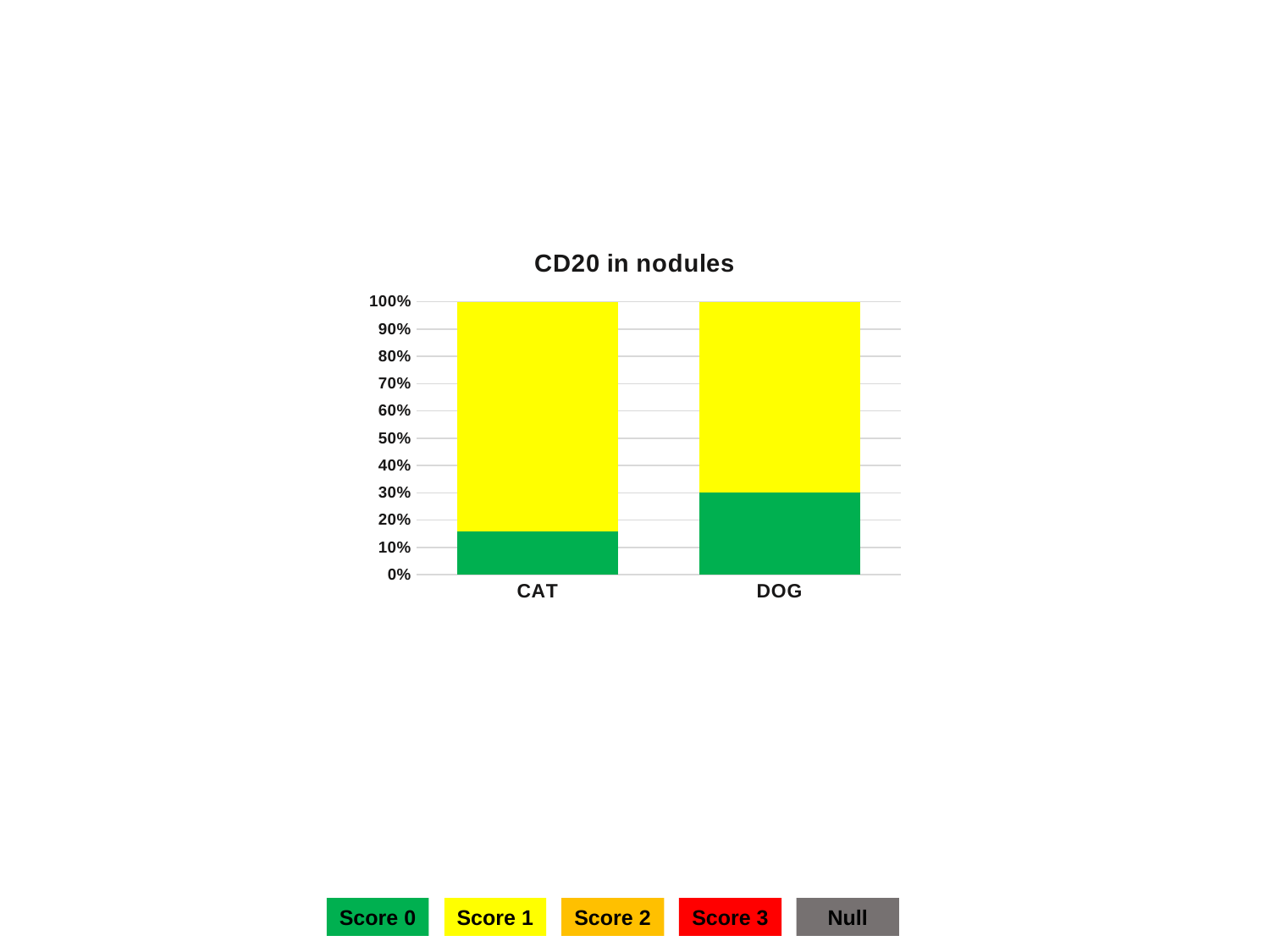

### Chart: CD20 in nodules
| Category | 0 | 1 | 2 | 3 | nd |
|---|---|---|---|---|---|
| CAT | 3.0 | 16.0 | None | None | None |
| DOG | 3.0 | 7.0 | None | None | None |Null
Score 2
Score 3
Score 0
Score 1
