# Supplementary material for: Feline and Canine Cutaneous Lymphocytosis: Reactive Process or Indolent Neoplastic Disease?
Source: Vet Sci. 2022 Jan 11;9(1):26. doi: 10.3390/vetsci9010026 (PMC8778986; doi:10.3390/vetsci9010026)
Supplement: Supplementary file 1 [file vetsci-09-00026-s001.zip › Supplementary tables.pdf]

Supplementary Table 1. Primer nucleotide sequences for PARR assay.

PARR	Receptor	Primer name	Sequence	Product size (bp)	Reference
Cat	BCR	V3F1	5'-GGTGGAGTCTGGGGGAGACCTG-3'	310-380	Mochizuki et al., 2011
		V3F2	5'-GGGGGTCCCTGAGACTCACCTG-3'	270-340	
		V3F3	5'-GGGTCCGCCAGGCTCCAGG-3'	210-280	
		V3F4	5'-GGCCGATTCACCATCTCCAGAGAC-3'	120-190	
		JR1	5'-GCYSTCACCAGGRYTCCYBGGC -3'	common reverse V3F1-4	
		JR2	5'-GCTGYGACHMTDGTTCCAYGGCCC-3'		
		JR3	5'-GCGRTGAYCWGGGTRYCYTGGC-3'		
		JR4	5'-GCGGTGACCAGGGTCCCGGGCCC-3'		
		JR5	5'-GCCGTCACCAGGGTTCCGACGCC-3'		
	TCR	TCRG V	5'-AAGAGCGAYGAGGGMGTGT-3'	80-120	Moore et al., 2005
		TCRG J	5'-CTGAGCAGTGTGCCAGSACC-3'		
Dog	BCR	Cμ 1	5'-TTCCCCCTCATCACCTGTGA-3'	130	Burnett et al., 2003
		Cμ 2	5'-GGTTGTTGATTGCACTGAGG-3'		
		CB1	5'-CAGCCTGAGAGCCGAGGACAC-3'	85-139	
		CB2	5'- TGAGGAGACGGTGACCAGGGT-3'		
		CB1	5'-CAGCCTGAGAGCCGAGGACAC-3'	about 120	
		CB 3	5'-TGAGGACACAAAGAGTGAGG-3'		
	TCR	TCRγ1	5'-ACCCTGAGAATTGTGCCAGG-3'	about 90	
		TCRγ2	5'-GTTACTATAAACCTGGTAAC-3'		
		TCRγ3	5'-TCTGGGVTGTAVTACTGTGCTGTCTGG-3'		

Supplementary Table 2. Score assessment of histological and immunohistochemical findings in cases of feline lymphocytosis.

Case number	Epidermis									Adnexa			Dermis involvement						Cellular infiltrate								IHC			
	Crust (0-1)	Ulcer (0-1)	Basket weave hyperkeratosis (0-3)	Compact hyperkeratosis (0-3)	Parakeratosis (0-3)	Hyperplasia (0-3)	Spongiosis (0-3)	Inflammation (0-3)	Epidermotropism (0-3)	Folliculotropism (0-3)	Siringotropism (0-3)	Sebaceous gland tropism (0-3)	Superficial (0-3)	Mid (0-3)	Deep (0-3)	Perivascular (0-3)	Diffuse (0-3)	Grenz zone (0-1)	Small lymphocytes (0-1)	Medium lymphocytes (0-1)	Large lymphocytes (0-1)	Indented lymphocytes (0-1)	Lymphocyte aggregates (0-1)	Mitosis (x 10 HPF)	Eosinophils (0-3)	Mast cells (0-3)	CD3 infiltrates (0-3)	CD20 infiltrates (0-3)	CD3 in nodules (0-1)	CD20 in nodules (0-1)
**1**	1	1	1	0	0	1	0	0	0	1	0	nd	1	2	3	3	3	0	1	1	0	0	3	0	1	1	3	0	0	1
**2**	0	0	0	1	0	2	0	0	1	nd	nd	nd	3	2	3	3	3	0	1	1	0	1	0	0	0	1	3	0	0	1
**3**	1	1	1	0	0	2	0	0	0	0	0	nd	3	3	3	3	3	1	1	1	0	1	1	0	0	1	3	0	0	1
**4**	1	1	0	0	0	3	1	0	1	0	0	nd	2	1	1	3	3	0	1	1	0	1	1	0	0	1	3	0	0	1
**5**	0	0	0	0	0	2	0	0	0	0	nd	nd	3	3	3	3	3	0	1	0	0	1	0	0	0	0	3	0	0	0
**6**	0	0	0	0	0	2	0	0	1	0	0	nd	3	2	1	0	3	0	1	0	0	1	0	0	0	0	3	0	0	1
**7**	1	0	2	0	0	2	0	0	1	1	nd	nd	3	3	3	3	3	0	1	1	0	0	0	0	1	0	3	0	0	1
**8**	0	0	1	0	0	2	0	1	2	2	1	nd	2	1	3	2	3	0	1	0	0	1	0	0	0	2	3	1	0	1
**9**	0	0	1	0	0	2	0	0	0	1	0	0	3	3	3	3	3	1	1	1	0	1	0	2	0	0	3	0	0	1
**10**	1	0	0	0	0	2	0	0	1	2	nd	nd	3	3	3	3	3	0	1	1	0	1	0	1	0	1	3	0	1	1
**11**	1	1	0	0	0	2	1	0	1	2	0	0	1	3	3	3	3	0	1	0	0	1	0	0	0	1	3	0	0	1
**12**	0	0	1	0	0	2	0	0	0	0	0	0	3	3	nd	3	3	1	1	0	0	1	1	0	3	1	3	0	0	1
**13**	0	0	1	0	0	2	0	0	0	0	nd	0	3	2	nd	3	3	0	1	1	0	0	0	0	0	0	3	0	0	1
**14**	1	0	0	1	1	2	1	0	1	1	nd	nd	2	2	1	3	2	0	1	1	0	1	1	0	0	1	3	0	0	1
**15**	0	0	0	0	0	2	0	0	0	nd	nd	nd	3	3	3	3	3	0	1	1	0	0	0	26	0	0	3	0	0	0
**16**	0	0	1	0	0	1	0	0	0	0	nd	nd	3	3	3	3	3	1	1	1	0	1	1	0	0	0	3	0	0	1
**17**	1	1	3	3	0	2	0	0	1	1	0	nd	3	1	2	3	3	0	1	0	0	1	0	0	0	1	3	0	1	1
**18**	0	0	1	2	0	2	1	0	2	0	0	0	3	3	3	3	3	0	1	0	0	0	1	0	0	1	3	0	0	1
**19**	1	0	3	1	0	2	0	0	0	1	0	nd	1	2	1	1	2	1	1	1	0	1	0	0	0	2	3	0	0	0

HPF=high power field; IHC=immunohistochemistry; nd=non detectable (structures not present within the examined section).

Supplementary Table 3. Score assessment of histological and immunohistochemical findings in cases of canine lymphocytosis.

	Epidermis									Adnexa			Dermis involvement						Cellular infiltrate								IHC			
**Case number**	Crust (0-1)	Ulcer (0-1)	Basket weave hyperkeratosis (0-3)	Compact hyperkeratosis (0-3)	Parakeratosis (0-3)	Hyperplasia (0-3)	Spongiosis (0-3)	Inflammation (0-3)	Epidermotropism (0-3)	Folliculotropism (0-3)	Siringotropism (0-3)	Sebaceous gland tropism (0-3)	Superficial (0-3)	Mid (0-3)	Deep (0-3)	Perivascular (0-3)	Diffuse (0-3)	Grenz zone (0-1)	Small lymphocytes (0-1)	Medium lymphocytes (0-1)	Large lymphocytes (0-1)	Indented lymphocytes (0-1)	Lymphocyte aggregate (0-1)	Mitosis (x 10 HPF)	Eosinophils (0-3)	Mast cells (0-3)	CD3 infiltrates (0-3)	CD20 infiltrates (0-3)	CD3 in nodules (0-1)	CD20 in nodules (0-1)
**1**	1	0	1	0	0	1	0	0	1	no	nd	0	3	3	nd	3	3	0	1	0	0	1	0	0	1	1	3	0	0	0
**2**	0	1	1	1	0	2	0	0	0	1	0	0	1	3	3	3	3	0	1	1	0	1	1	0	1	0	3	0	1	1
**3**	0	0	2	3	3	2	0	0	1	0	3	nd	3	3	1	3	3	0	1	1	0	1	1	1	0	1	3	0	1	1
**4**	1	0	2	0	0	2	0	0	1	0	0	0	3	1	2	3	3	0	1	1	0	1	0	0	0	0	0	0	0	0
**5**	0	0	2	2	0	2	1	1	2	0	0	0	2	3	2	3	3	0	1	1	0	1	2	2	0	0	3	1	0	1
**6**	1	0	3	0	0	2	0	0	0	0	1	nd	3	1	0	3	3	0	1	1	0	1	0	1	1	1	1	3	0	0
**7**	0	0	3	0	0	2	0	0	2	0	1	nd	1	2	2	3	3	0	1	1	0	0	1	0	2	1	3	2	0	1
**8**	0	0	0	0	0	1	0	0	0	1	0	0	2	2	2	3	3	0	1	0	0	1	1	1	2	1	2	3	1	1
**9**	0	0	3	0	0	1	0	0	2	0	0	0	3	2	0	3	3	0	1	1	0	0	0	0	1	1	3	0	0	1
**10**	1	0	3	3	3	3	0	1	1	0	0	0	2	1	1	1	3	0	1	1	0	1	1	0	0	2	3	2	0	1

HPF=high power field ; IHC=immunohistochemistry; nd=non detectable (no structures present within the examined section).
